# Factors determining the sensitivity to proteasome inhibitors of multiple myeloma cells

**DOI:** 10.3389/fphar.2024.1351565

**Published:** 2024-03-04

**Authors:** Marta Pelon, Patryk Krzeminski, Zuzanna Tracz-Gaszewska, Irena Misiewicz-Krzeminska

**Affiliations:** ^1^ Department of Experimental Hematology, Institute of Hematology and Transfusion Medicine, Warsaw, Poland; ^2^ Department of Nanobiotechnology, Biology Institute, Warsaw University of Life Sciences, Warsaw, Poland

**Keywords:** proteasome, proteasome inhibitors, bortezomib, carfilzomib, multiple myeloma

## Abstract

Multiple myeloma is an incurable cancer that originates from antibody-producing plasma cells. It is characterized by an intrinsic ability to produce large amounts of immunoglobulin-like proteins. The high rate of synthesis makes myeloma cells dependent on protein processing mechanisms related to the proteasome. This dependence made proteasome inhibitors such as bortezomib and carfilzomib one of the most important classes of drugs used in multiple myeloma treatment. Inhibition of the proteasome is associated with alteration of a number of important biological processes leading, in consequence, to inhibition of angiogenesis. The effect of drugs in this group and the degree of patient response to the treatment used is itself an extremely complex process that depends on many factors. At cellular level the change in sensitivity to proteasome inhibitors may be related to differences in the expression level of proteasome subunits, the degree of proteasome loading, metabolic adaptation, transcriptional or epigenetic factors. These are just some of the possibilities that may influence differences in response to proteasome inhibitors. This review describes the main cellular factors that determine the degree of response to proteasome inhibitor drugs, as well as information on the key role of the proteasome and the performance characteristics of the inhibitors that are the mainstay of multiple myeloma treatment.

## 1 Introduction

The proper functioning of any human cell requires the coordination of the functions of numerous cellular structures, organelles, and complexes. Cells do not “waste” and require continuous recycling and removal of damaged or unwanted proteins. This key biological process is provided by the ubiquitin-proteasome system (UPS), which is a line of defense against toxic proteins. The ubiquitin-proteasome system is a major proteolytic pathway that regulates the state of the proteome during cell development and aging. It targets proteins that regulate cellular processes such as gene expression, gene stress response and cell cycle progression (Ciechanover, 2009; Chowdhury and Enenkel, 2015). Importantly, the proteasome does not destroy random proteins, but those labeled by the cell in the so-called ubiquitination process, which involves the covalent attachment of ubiquitin (Ub) to a substrate protein, with the formation of a polyubiquitin chain. The attachment of ubiquitin to a protein is a three-step process. The activating enzyme E1 transfers ubiquitin to the carrier E2 where, with the help of the ligase E3, it is permanently attached to the protein. Because ubiquitin activation occurs in the presence of ATP, the first step of the reaction is controlled by a class of ATP-dependent proteases. Subsequent steps are performed in turn by proteases that are representatives of the ATP-independent class. The reaction catalyzed by a specific E3 ligase leads to the final attachment of ubiquitin to the lysine in the target protein. The attachment of subsequent ubiquitin molecules leads to the formation of a polyubiquitin chain. The elongation process results in the final targeting of the tagged protein to the 26S proteasome responsible for the degradation of cytosolic proteins in Eukaryota (Ciechanover, 2005). The degradation pathway of cytosolic proteins in mammals leads to the production of peptides of 5–18 amino acids which are then transported to the endoplasmic reticulum where they are presented on the surface of MHC I proteins (Nandi et al., 2006).

DUB deubiquitinating enzymes (DUBs) can reverse these effects by cleaving the peptide bond between ubiquitin and its substrate protein. Thus, they play antagonistic role to ligases, removing ubiquitin and thus reversing the fate of proteins (Komander, Clague, and Urbé, 2009; Komander, 2010; Farshi et al., 2015). Deubiquitinases belonging to the UCH (Ubiquitin C-terminal Hydrolase) family act on small ubiquitin molecules attached to proteins by processing them and severing them from the polyubiquitin chain. Although the process of protein deubiquitination is not as well understood as ubiquitination, it is known that some DUB enzymes are involved in a broad spectrum of biological processes (D.-H. Wang et al., 2018). In turn, abnormal function of the enzymes of this group of UPS may contribute to numerous pathological conditions (Farshi et al., 2015). In an effort to maintain protein homeostasis, cells must not only actively control protein production, but also protein degradation. The attachment of ubiquitin to the protein of interest can affect the interaction, localization, and most importantly, the timing and extent of protein degradation within the cell (Jara, Frank, and Özdinler, 2013). Numerous processes rely on proteasome function, making it a crucial target for therapeutic intervention in many diseases (Schmidt and Finley, 2014).

## 2 Proteasome structure and functions

A proteasome is a protein complex whose components exhibit distinct enzymatic properties. In eukaryotic cells, proteasomes are found in 2 basic forms: 20S and 26S.

The main component of the UPS in eukaryotic cells is proteasome 26S which is a 2.4-MDa molecular machine (Lo, Cheng, and Klein, 1981). The 26S proteasome catalyzes protein degradation in mammalian cells, including the rapid degradation of misfolded and regulatory proteins and most of the slower breakdown of the bulk of cellular proteins (Collins and Goldberg, 2017). The structure of the 26S proteasome consists of a 20S catalytic core and a 19S regulatory complex.

**FIGURE 1:**
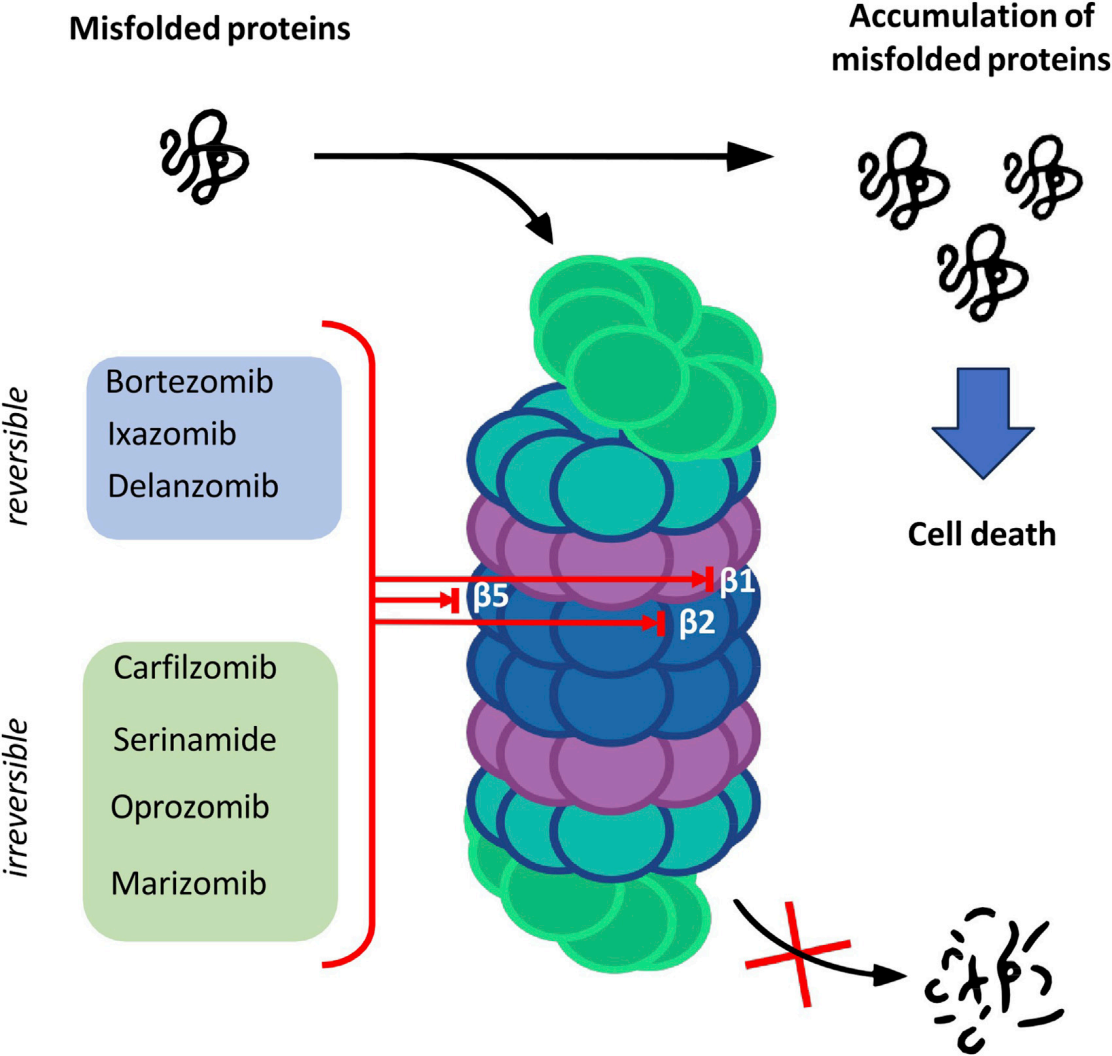
The overview of the proteasome structure and functions, and targets of proteasome inhibitors.

The catalytic core is built of 4 rings forming a cylindrical structure. Each ring consists of 7 subunits—outer rings are alpha subunits, whereas inner rings are formed by beta subunits. Catalytic sites are located on the inner surface of the 20S proteasome cylinder where protein substrates are bound. The N-terminal peptide extension of the eukaryotic α3 subunit is the key contributor to the α-ring gate which is mostly closed in the absence of activators (Osmulski, Hochstrasser, and Gaczynska, 2009). In the case of β-ring only three subunits of the core show enzymatic activity: β1, β2 and β5 which are responsible for caspase-like, trypsin-like and chymotrypsin-like activities, respectively (Budenholzer et al., 2017). The aforementioned activities presented by subunits of the proteasome core are targeted by proteasome inhibitors (PIs) which have a major impact on the treatment of hematologic malignancies such as multiple myeloma (MM) and mantle cell lymphoma (Nunes and Annunziata, 2017) (Figure 1).

The second part of the 26S proteasome is an activator 19S that consists of the lid-an eight-subunit subcomplex of the regulatory particle (RP) and the base which contains the six proteasomal ATPases: Rpt1–Rpt6, and four non-ATPase subunits: Rpn1,Rpn2, Rpn10, and Rpn13. 19S regulatory molecule controls the access to active proteolytic sites. This regulatory complex is responsible for shutting down one or two ends of the 20S core peptidase carrying substrates into the degradation compartment (Bard et al., 2018).

## 3 Immunoproteasome structure and signal leading to its formation

In most cells, under the influence of appropriate stimulation, the constitutively produced proteasome is converted into immunoproteasome. This type of proteasome is constitutively expressed in hematopoietic cells and induced in non-immune cells following exposure to tumor necrosis factor (TNF) and proinflammatory cytokines such as IFN-α, IFN-β, or IFN-γ (Murata et al., 2018). The three standard catalytically active subunits of core β1, β2, and β5 are replaced by β1i (LMP2), β2i (MECL-1), and β5i (LMP7) whereas regulatory part of 19S is replaced by 11S regulatory structure (Crawford, Walker, and Irvine, 2011).

The proteasome isoform generated by IFN-γ was termed the immunoproteasome to emphasize its role in processing intracellular antigens for presentation on MHC I. The rapid response to the immune stimuli is ensured by the rate of folding of the immunoproteasome, which is four times faster than that of the constitutive proteasome (Murata et al., 2018)).

## 4 Thymoproteasome-structure and function

Thymoproteasome is a form of proteasome exclusively expressed in the thymus, and was found in both human and mouse (Murata et al., 2007). Particularly, β5t is expressed exclusively in cortical thymic epithelial cells (cTECs), which are responsible for the positive selection of developing T cells in the thymus. It is involved in CD8^+^ T cell development (Murata, Takahama, and Tanaka, 2008) In thymoproteasome a sequence proximal to the Psmb5 locus encoding the β5 subunit encodes a protein termed β 5t (PSMB11). β 5t is structurally homologous to β 5 and β 5i (PSMB8) but contains many hydrophilic amino acids in the substrate pocket in contrast to β 5 and β 5i consisting mainly of hydrophobic amino acids. This allows the cells to exhibit unique substrate specificity in endopeptidase proteolysis leading to a unique set of peptides presented on MHC class I. (Murata et al., 2018).

## 5 Proteasome inhibitors

Proteolysis is a fundamental metabolic process and complete blockade of the proteasome activity with an inhibitor results in the cell death. The table below shows the classification of proteasome inhibitors grouped by structural class and type of binding to proteasome subunits (Tab.1) (Demo et al., 2007; Oerlemans et al., 2008; Tan et al., 2021).

**TABLE 1:** Proteasome inhibitors in clinical practice and development.

Structural class	Proteasome inhibitor	Binding	Target
Peptide Boronic acid	Bortezomib	reversible	CT-L, T-L, C-L
	Delanzomib	reversible	CT-L
	Ixazomib	reversible	CT-L
Peptideepoxyketone	Carfilzomib	irreversible	CT-L, T-L, C-L
	Serinamide	irreversible	CT-L
	Oprozomib	irreversible	CT-L
β-lactone	Marizomib	irreversible	CT-L, T-L, C-L

CT-L, chymotrypsin-like; T-L, trypsin-like; C-L, caspase-like.

### 5.1 Peptide boronic acids

Owing to the covalent reversible binding to amino acids or sugars, peptide boronic acids can be widely used as covalent reversible enzyme inhibitors glycan and RNA carriers or ligands. Over the past decade, many peptide boronic acids have been developed as inhibitors of various enzymes, including threonine proteases, serine proteases, aspartyl proteases, and arginases. In addition, the significant potential of peptide boronic acids is confirmed by the presence of bortezomib used in the treatment of MM (Tan et al., 2021).

### 5.2 Peptideepoxyketone

Peptide α′,β′-epoxyketones which are based on the natural product epoxomicin demonstrate anti-inflammatory and antitumor activity through inhibition of the proteasome. Compounds belonging to this group of inhibitors show specificity and selectivity. The specificity of peptidoepoxyketone is connected with their epoxyketone pharmacore which forms an unusual six-membered morpholino ring with the amino terminal catalytic Thr-1 of the 20S proteasome (Bo Kim, Fonseca, and Crews, 2005).

### 5.3 β-lactone

β-lactone is a non-peptidic class of proteasome inhibitors. All three proteasomal activities can be irreversibly inhibited by the β-lactone ring (Lawasut et al., 2012). This class of PI inhibits the proteasome through reaction with the hydroxyl group on the active site threonine to form an acyl enzyme conjugate (Adams, 2003).

## 6 Proteasome inhibitors in multiple myeloma

Multiple myeloma is an incurable hematologic malignancy. It is a cytogenetically heterogeneous clonal plasma cell proliferative disorder characterized by high amount of monoclonal protein produced by UPS dysregulations (Rajkumar et al., 2014). The development of proteasome inhibitors has improved the prognosis of patients and two of them appear to play a key role in the fight against MM.

### 6.1 Bortezomib

Bortezomib (Velcade™, PS-341) belonging to the Peptide Boronic acid class, is the first proteasome inhibitor approved by the Food and Drug Administration for the treatment of patients with progressive MM (Kane et al., 2006). Bortezomib also exhibits activity in other lymphoproliferative disorders such as mantle cell lymphoma, Waldenstrom macroglobulinemia or T-cell lymphoma amyloidosis (Robak and Robak, 2019). In addition to its action on the MM cell, bortezomib also exhibits effects on the bone marrow microenvironment. It contributes to inhibiting the adhesion of MM cells to the bone marrow stromal cells which increases sensitivity of MM cells to apoptosis and also exhibits anabolic effects on the bone (Curran and McKeage, 2009). Bortezomib interrupts prosurvival paracrine and autocrine cytokine loops in the bone marrow microenvironment mediated by interleukin-6 (IL-6), vascular endothelial growth factor (VEGF), insulin-like growth factor 1 (IGF-1), and tumor necrosis factor-α (TNF-α) (Hideshima et al., 2003; Curran and McKeage, 2009). The main mechanism of action is based on the inhibition of the proteosomal degradation of NFκB inhibitor IκB which consequently leads to the retention of the nuclear factor kappa B (NFκB) complex in the cytoplasm. Prevention of NFκB translocation to the nucleus results in the inactivation of downstream pathways crucial for MM cell signaling (Field-Smith, Morgan, and Davies, 2006).

Bortezomib induces changes in transcripts involved in the regulation of apoptosis, proteasome function, cell growth and heat shock response (Mitsiades et al., 2002; Mitsiades et al., 2003). Another mechanism associated with the anticancer properties of bortezomib appears to be a proapoptotic member of the Bcl-2 protein family NOXA. This proapoptotic factor is induced by bortezomib in cancer cells. Upregulation of NOXA induces apoptosis by interacting with anti-apoptotic proteins (Chen et al., 2011).

### 6.2 Carflizomib

Carfilzomib is a second-generation proteasome inhibitor with similar proapoptotic effects to bortezomib. They both initiated apoptosis through extrinsic pathway mediated by activation of Fas/caspase-8-dependent signaling and an intrinsic pathway involving cytochrome c release and caspase-9 activation (Kuhn et al., 2007).

This cell-permeable tetrapeptide epoxyketone analog of epoxomicin forms stable and irreversible adducts exclusively with the proteasome. Carfilzomib induced a dose- and time-dependent inhibition of proliferation leading to programmed cell death. Apoptosis in this case was associated with mitochondrial membrane depolarization, activation of caspase pathways and c-Jun-N-terminal kinase (JNK) (Ziogas et al., 2017).

## 7 Sensitivity to proteasome inhibitors

**FIGURE 2:**
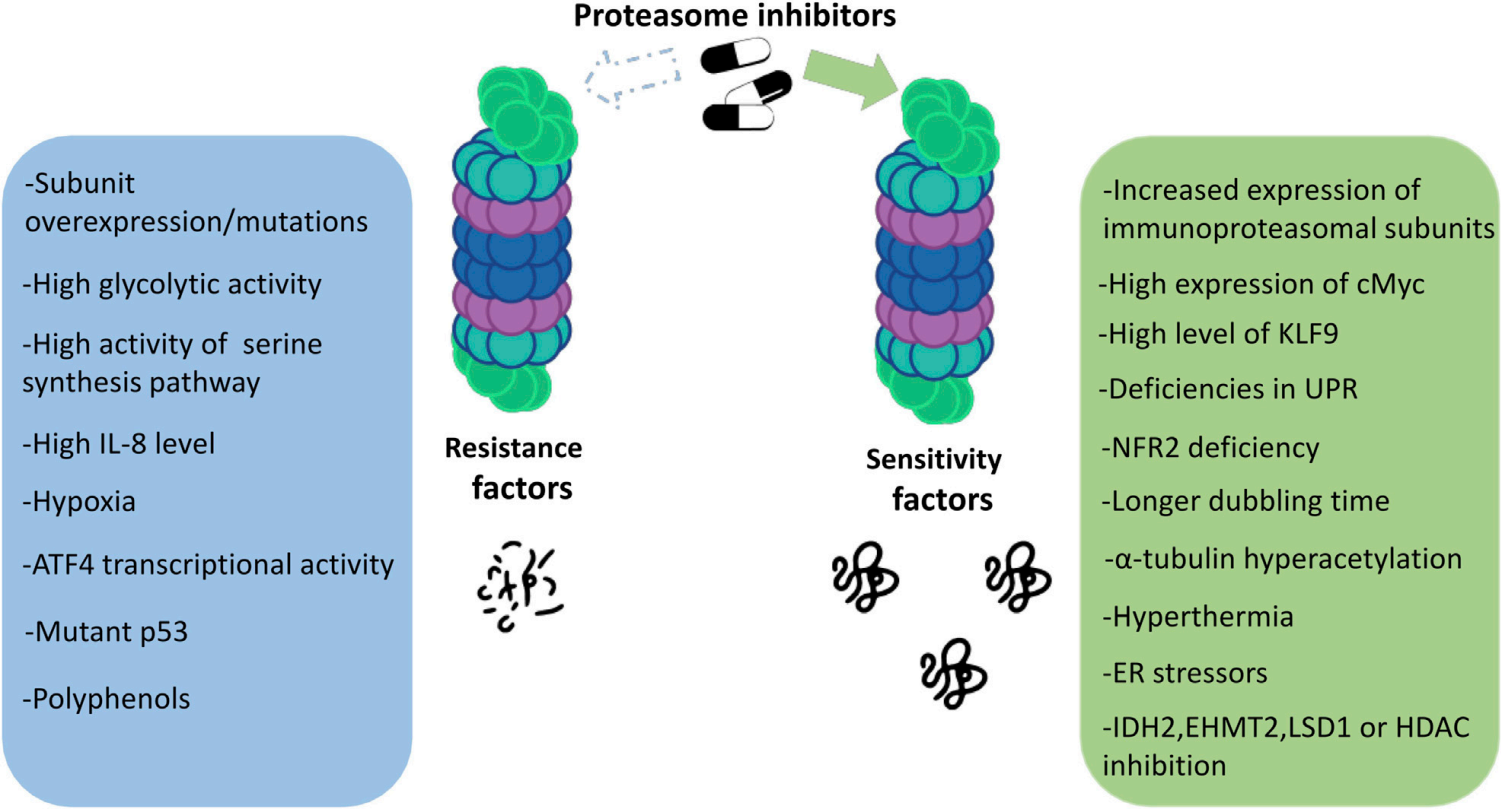
Factors determining the sensitivity to proteasome inhibitors in multiple myeloma.

Proteasome inhibitors are known to be effective against MM. Drugs from this group show unique features compared to other anticancer drugs. Although we now have a great deal of knowledge about PIs, all the mechanisms of their action and also the susceptibility to their effects on patients still remain unclear. Which mechanisms are responsible for the responsiveness of proteasome inhibitors and what determines the strength of the response to a given drug? The answers to these questions are still in the realm of research and speculation. Some of the main factors determining the degree of response to proteasome inhibitors are discussed below (Figure 2).

### 7.1 Transcription factors

The functional properties of cells are dependent on gene expression, in response to physiological, developmental and environmental stimuli. Central to maintaining the cell’s adaptive capacity is the coordinated regulation of gene transcription. This process is mastered by proteins called transcription factors (TFs). They are involved in controlling gene activity profiles and regulating a wide variety of cellular functions by interacting directly with DNA and with non-DNA-binding accessory proteins (Fulton et al., 2009).

One of the transcriptional factors which plays a crucial role in organism development, cell differentiation, and even apoptosis is cMyc. However, when deregulated, c-Myc becomes an oncogene that is implicated in the development of malignant cells. C-Myc is one of the factors influencing the response rate to proteasome inhibitors. It turns out that it interacts with proteasome. The interplay between proteasome, the proapoptotic protein NOXA, and the c-Myc oncogene provides an explanation for the selectivity of proteasome inhibitors against cancer cells with deregulated c-Myc (Nikiforov et al., 2007).

Another tumor suppressor-p53 restricts proliferation of genetically damaged cells by the induction of permanent cell cycle arrest or senescence, or activation of cell death when cell damage is too severe (Nikiforov et al., 2007). P53-mediated apoptosis is the mechanism of cellular toxicity induced by bortezomib (Xue et al., 2023). In newly diagnosed MM, TP53 mutations are present in 3% of patients and its frequency increases at further stages of malignancy and represents a marker of progression (Lionetti et al., 2016). Mutated p53 can disrupt the apoptotic pathway that is normally activated in response to proteasome inhibition. This can lead to reduced sensitivity of cancer cells to the cytotoxic effects of proteasome inhibitors, thus contributing to drug resistance. Thus, the presence of mutated p53 in the cells of advanced MM may increase resistance to PI. Accumulation and phosphorylation of p53 caused by proteasome inhibition leads to negative regulation of the cell cycle and activates pro-apoptotic proteins including NADPH oxidase activator (NOXA) and Bcl-2-associated X protein (Bax), resulting in apoptosis via mitochondrial pathway (Jayaweeraet al. 2021). Additionally, treatment of MM TP53-WT cell lines with Nutlin, which interferes with MDM2-p53 interaction, releases p53 from negative control and thereby enhances cytotoxicity of bortezomib (Saha et al., 2010).

Nuclear factor-kappa B (NF-κB) is a transcription factor that regulates inflammatory and immune responses, as well as cancer cell proliferation and survival, angiogenesis, and the tumor-microenvironment crosstalk. Many first-line anti-MM drugs affect NF-κB signaling pathway. As a reversible inhibitor of the 26S proteasome, bortezomib suppresses NF-κB activation by preventing its proteasomal cleavage and degradation of its repressor IκB kinase (Bu et al., 2014)e. The inhibitory effect on NF-κB signaling was initially the main reason for the use of bortezomib in the treatment of cancer (Vrábel, Pour, and Ševčíková, 2019). Even with next-generation PIs such as carfilzomib and ixazomib, the NF-κB pathway is a primary or secondary target in MM therapy (Vrábel, Pour, and Ševčíková, 2019). Studies with ixazomib on MM cells showed that inhibition of NF-κB signaling results in reduced production of cytokines and growth factors promoting MM cells growth and progression (Chauhan et al., 2011). However, Hideshima et al. propose that bortezomib not only inhibits the activity of inducible NF-κB but also its impact on constitutive NF-κB in MM cells is more likely to be the opposite. The authors present that bortezomib induces IKKβ-dependent downregulation of IκB, thereby activating NF-κB signaling. Activation of NF-κB by bortezomib may be repressed by tumor necrosis factor receptor super family member 18 (TNFRSF18, also called GITR) in MM cells through the suppression of NF-κB translocation to the cell nucleus (Hideshima et al., 2009). GITR has been identified recently as a tumor suppressor in MM and its expression correlates positively with the response of MM cells to bortezomib (Zhao et al., 2015). Thus, NF-κB - dependent effects of PIs treatment on cancer cell survival may depend of cell type, stage of disease, and the cellular context.

HIF-1α is a master transcriptional regulator of cellular response to hypoxia. It is often upregulated in cancer cells, through both hypoxic and non-hypoxic pathways, to promote cancer cell proliferation, metabolic reprogramming, angiogenesis, and cancer cell stemness (Wicks and Semenza, 2022). HIF-1α activation promotes cancer cell survival and leads to chemo- or radioresistance through modulation of cellular metabolism, increased ability of DNA repair, inhibition of apoptosis, and stimulation of autophagy (Xia, Jiang, and Zhong, 2018). However, the contribution of HIF-1α in the response to PIs in MM is complex. On the one hand, PIs can lead to the stabilization and accumulation of HIF-1α through blocking its degradation. This stabilization of HIF-1α can promote the survival of MM cells by upregulating pro-survival and anti-apoptotic genes. In accordance with these observations, stable inhibition of HIF-1α in MM cells suppressed proliferation and sensitized cells to the therapeutic effect of bortezomib (Maiso et al., 2015). On the other hand, some studies have suggested that the corresponding increased level of HIF-1α was inactive. For example, Shin et al., 2008 present that bortezomib represses the transcriptional activity of HIF1-α by inhibiting the recruitment of its coactivator p300. Interestingly, bortezomib may attenuate HIF-1- but not HIF-2-mediated transcriptional activation (Abd-Aziz, Stanbridge, and Shafee, 2015).

Signal transducers and activators of transcription (STAT) is a family of transcription factors critical for cancer cell proliferation, angiogenesis, invasion, metastasis, and increased survival. Activated by growth factor receptors and oncogenic kinases such as epidermal growth factor receptor (EGFR), Janus associated kinases (JAK) or Src family kinases, they induce oncogenic response by regulating gene expression. Among seven members of STAT family, STAT3 and STAT5 are most strongly involved in cancer pathogenesis (Chong, Chng, and de Mel, 2019). In MM, STAT3 expression has been shown to be an adverse prognostic factor that may promote drug resistance (Jung et al., 2017). In MM cells, STAT3-dependent expression of PSMB6 that constitutes the caspase activity of the proteasome also appears to be crucial for bortezomib resistance, as STAT3 inhibitor Stattic overcomes bortezomib-resistance in MM by reducing PSMB6 (Yuan et al., 2023). Additionally, *in vitro* synergy of STAT3 inhibitors such as ruxolitinib, INCB16562, hydrocalamenene, genipin, and Iiariside II with bortezomib has been demonstrated in MM cells, via various mechanisms including JAK or SRC inhibition, or PIAS3, SHP1, and PTEN upregulation (Chong, Chng, and de Mel, 2019). Finally, Zhang et al. propose a regulatory pathway in which tight junction protein 1 (TJP1) inhibits EGFR/JAK1/STAT3 signaling, possibly through a direct interaction between TJP1 and EGFR, in MM cells. Suppressed EGFR/JAK1/STAT3 signaling results in decreased proteasome capacity through reduced expression of the catalytic subunits LMP7 and LMP2, thereby increasing PI sensitivity. Thus, the authors propose TJP1 as a biomarker of bortezomib sensitivity, however, the results of pre-clinical studies suggest its possible role in response to other PIs (X.-D. Zhang et al., 2016).

Proteasome inhibition activates ER stress resulting in increased expression of ATF4 followed by ATF3 upregulation. Heterodimerization of these two proteins promotes upregulation of pro-apoptotic factors. In MM cells, ER stress and subsequent unfolded protein response (UPR) are the main mechanisms triggering PI-induced apoptosis (Obeng et al., 2006). Lower expression of ATF3 and ATF4 correlates with shorter progression-free survival in MM patients receiving bortezomib combined with dexamethasone (Narita et al., 2015).

The nuclear factor erythroid 2-related factor 2 (NRF2) is one of the key components of the cellular response to oxidative stress. In MM, NRF2 is constitutively activated in about half of the primary samples and proteasome inhibition induces its further activation. Going further, its deficiency significantly increases sensitivity of MM cells to bortezomib and carfilzomib through increased ER-stress induced apoptosis (Sun et al., 2016)**.** Additionally, NRF2 contributes to the adaptive resistance to proteasome inhibitors via increased expression of proteasome (Li et al., 2015).

The correlation of increased NRF2 activation with tumor progression and aggressiveness has been thoroughly reviewed by Choi et al. (Choi, Kim, and Kwak, 2021) For example, NRF2 status was significantly associated with histological grade of breast cancer (Onodera et al., 2014) or its expression correlates with the progression of gastric cancer (Kawasaki et al., 2015).

Additionally, as shown in colon cancer cells (Arlt et al., 2009) NRF2-dependent expression of the proteasome’s components translates into higher proteasome activity. Pharmacological activation of NRF2 increases the expression of several proteasome subunits and thus increases proteasome activity in human fibroblasts (Kapeta, Chondrogianni, and Gonos, 2010). In MM cell lines that exhibit reduced activity of PIs the restoration and increased synthesis of the β5 subunit of the proteasome was observed as a result of acute accumulation of NRF2 (Sogabe et al., 2024).

Oxidative stress, proteasome inhibition, or heat shock causes the failure of UPS which results in the formation of aggresomes. These subcellular structures that contain misfolded proteins, heat-shock proteins, and the components of the proteasome, deliver aggregated proteins to lysosomal degradation (Corboy, Thomas, and Wigley, 2005)**.** In MM cells bortezomib treatment induces aggresomes formation, which was associated with apoptosis (Catley et al., 2006).

### 7.2 Oxidative stress

The oxidative stress is exerted by reactive oxygen species (ROS) that accumulate in the cell as a result of an imbalance between ROS generation and elimination by antioxidants. Cancer cells produce higher amounts of ROS than normal cells due to the alterations in the signaling pathways that affect metabolism (Gorrini, Harris, and Mak, 2013)**.** Proteasome inhibitors have been associated with inducing oxidative stress in cells. They prevent the degradation of ubiquitinated proteins, leading to the accumulation of misfolded or damaged proteins within the cell. This accumulation leads to the endoplasmic reticulum (ER) stress which, in turn, may lead to the generation of ROS as part of the UPR (Lipchick, Fink, and Nikiforov, 2016).

Macrophage migration inhibitory factor (MIF) which acts as a chaperone for superoxide dismutase 1 (SOD1) mediates MM cell resistance to proteasome inhibitors by preventing ROS-induced mitochondrial dysfunction. Wang et al., 2020 shows that inhibiting MIF activity *in vivo* displayed synergistic antitumor activity with proteasome inhibitors and resensitized PI-resistant MM cells to treatment. The role in the response to PIs of the NRF2, which is one of the key components of cellular response to oxidative stress, has been described in Section 7.1.

### 7.3 Activity of proteasome subunits

Since the proteasomal system is responsible for the degradation of non-functional proteins, as well as for the cell’s response to oxidative stress, it is expected that cells possessing increased proteasome activity will have increased survival rates. Studies conducted on proteasome activity have shown that overexpression of the β5 subunit of the proteasome in various cell lines leads to an increase in the total proteasome activity, the expression level of proteasome subunit proteins and its efficient folding (Chondrogianni et al., 2005). Not only differences in the expression of gene encoding β5 subunit but also its mutations may result in the resistance to PIs. The upregulation of mutated PSMB5 leads to reduced proteasome activity which causes decrease in bortezomib binding (Balsas et al., 2012)**.**

Another issue is a type of presented proteasome. Immunoproteasomes are characterized by increased proteolytic activity, therefore differences in expression of proteasomal subunits may affect PI sensitivity (Busse et al., 2008). The results led to the conclusion that increased expression of immunoproteasomal subunits caused increase in proteasome activity and sensitization of cells to PIs.

The increased amount of assembled proteasome translates into a more functional proteasome, which provides enhanced survival after drug treatment, thus decreasing sensitivity to PIs.

### 7.4 E3 ligases

E3 ubiquitin ligases are a large family of enzymes that, together with the ubiquitin-activating enzyme E1 and the ubiquitin-coupling enzyme E2, combine to form a three-enzyme ubiquitination cascade. E3 ubiquitin ligases plays an important role in promoting ubiquitination and protein degradation. Importantly, ubiquitination modification is involved in almost all vital activities of eukaryotes.

Apparently, E3 ligases are also significantly involved in the processes promoting cancer progression such as proliferation, invasion, DNA damage and repair, metabolism, or regulation of apoptosis. As major regulators of protein homeostasis, E3 ligases are essential for the proper functioning of cells in various systems. According to the difference in structure and function, E3 ligases can be roughly divided into four types: HECT type, U-box type, RING-finger type, and RBR type (Q. Yang et al., 2021).

One of the genes included in the HECT E3 ligase group is NEDD4L. Downregulation of NEDD4L expression has been shown to reduce the sensitivity to bortezomib both *in vitro* and *in vivo*. This is due to the action of E3 ligase leading to increased autophagy of MM cells and decreased proteasome activity. In addition, the increase in NEDD4L expression has been shown to coincide with UPR activation and autophagy induced by ER stress-inducing drugs. NEDD4L is also responsible for the ubiquitination and degradation of ULK1 kinase involved in autophagy inactivation. It is well known that cancer cell activity depends on frequent changes between PI3K/AKT and PTEN, and it is pAKT-Ser473 that has been identified as the site of degradation of the NEDD4-1 tumor suppressor E3 ligase in many malignancies (Huang et al., 2022).

Another E3 ubiquitin ligase belonging to the RING-H2 type is p53-induced Pirh2. Downregulation of Pirh2 protein has been shown to be associated with an increase in nuclear factor-kappaB (NF-κB) p65, phospho-p65, pIKBa, and IKKa leading to decreased sensitivity to bortezomib. Pirh2 inhibits the NF-κB signaling pathway by inhibiting the phosphorylation and subsequent degradation of IKBa to overcome acquired bortezomib resistance in MM cells (L. Yang et al., 2018).

As is already known, both the occurrence and progression of cancer are accompanied by abnormalities in the ubiquitin system and E3 ligases may be involved in cellular responses to stress signaling induced by cancer development. Therefore, the creation and use of drugs targeting a specific E3 ligase may have better selectivity and less toxicity than the use of proteasome inhibitors that block degradation of entire proteins.

### 7.5 Immunoglobulin level

Multiple myeloma is characterized by the accumulation of immunoglobulin-secreting clonal plasma cells (PC) within the bone marrow (BM). It has been proposed that huge potential to produce immunoglobulins is a prerequisite for the exceptional sensitivity of MM cells to PIs (Meister et al., 2007). So far, classical hematological response criteria to the treatment are based on level of secreted serum monoclonal Ig (M-protein). M-protein, which is considered as a diagnostic hallmark of the all monoclonal gammopathies is one of the main values measured for every MM patient (Chim et al., 2018). Recent research proved that intracellular Ig (iIg) concentration represents the myeloma Ig production capacity more precisely than M-protein, that’s why quantification of iIg from aberrant plasma cells could serve as a better estimate of the immunoglobulins synthesis rate (Yan et al., 2020) Indeed, iIg is a robust, independent factor that can be used to predict the course of the disease in patients treated with the PI-based therapy (Vdovin et al., 2022).

### 7.6 Proteasome workload

Increased workload of proteasome in MM is connected to enhanced Ig production. It also appears to be connected with increasing production of rapidly degraded polypeptides (RDP). Not only abnormal proteins can be targeted for degradation, but also newly synthesized proteins. An imbalance between the degree of proteasome loading and its capacity leads to an accumulation of polyubiquitinated proteins. The aforementioned processes lead to a disruption of homeostasis, increasing the burden on proteasome and also activating the adaptive processes.

Initially, both the level of stress and sensitivity to proteasomal inhibition were correlated with the rate of cell proliferation. A decrease in the rate of proliferation led to a significant decrease in the level of polyubiquitinated proteins and the toxicity of proteasome inhibition. Thus, different toxicity of proteasome inhibitors is related mainly to stress conditions and adaptive responses (Bazzaro et al., 2006). However, when the doubling time of MM and solid tumor cell lines were directly compared, the interesting observation was made. MM cells, which have substantially longer cell population doublings, were actually more sensitive to the treatment. Thus, high cell proliferation, due to, i.e., higher turnover of proteasome-dependent cell-cycle proteins, does not make tumor cells more susceptible to proteasome inhibition (Borjan et al., 2019).

The main task of proteasome is disassembly of misfolded and damaged proteins. When this important machinery is damaged or disrupted, there is an accumulation of polyubiquitinated proteins leading eventually to cell death. Failure of protein folding, transport, or degradation can cause endoplasmic reticulum (ER) stress which is referred to as imbalance between the cellular demand for ER function and ER capacity. ER stress leads to the activation of UPR which is a highly conserved group of pathways that serve to restore ER homeostasis. It was shown that ER stress-induced proteasome overload resulted in sensitization of MM cells to proteasome inhibitor-induced apoptosis. Increased proteasome load induced by ER stressors led to a dramatic increase in sensitivity to PIs while increased proteasome activity resulted in the resistance to apoptosis induced by this group of drugs (Bianchi et al., 2009)

### 7.7 Epigenetic factors

Epigenetics regulates gene expression mainly through the changes in DNA methylation (at cytosines in CpG pairs), non-coding RNA expression, and posttranslational modification of histones. No changes in DNA sequence occur due to the epigenetics, however, epigenetic patterns can be maintained through the cell division providing long-lasting transcriptomic alternations. Various factors contribute to epigenetics including diet, exercise, extreme temperature, and exposure to pollutants. In MM, DNA methylation is important for the pathogenesis, progression, and prognosis of the disease. Previous studies reported general MM hypomethylation accompanied by hypermethylation of specific genes such glutathione peroxidase 3, *TP53* (Hurt et al., 2006), CDNK2B, DAPK (Dimopoulos, Gimsing, and Grønbæk, 2014), p15, and p16 (Ng et al., 1997). Not surprisingly the interplay between epigenetic and response rate to PI exists.

Decitabine, well known DNA methyltransferase inhibitor and hypomethylating agent, enhances bortezomib treatment efficacy in RPMI 8226 MM cells (Cao et al., 2016) and AML Kasumi-1 cell line (Mpakou et al., 2021). The possible mechanism of the observed phenomenon in MM is unknown. In AML bortezomib decreased Sp1 transcription factor (Sp1) protein levels and interfered with Sp1/NF-κB complex, leading to DNMT1 downregulation and transcription of methylation-silenced genes.

HDAC inhibitor such as approved by the FDA to treat non-refractory MM, panobinostat (LBH589), reduces cell growth and induces apoptosis in MM cells as a single agent, however, synergistic activity has been also observed in combination with bortezomib (Hideshima, Richardson, and Anderson, 2011). Possible mechanisms reach far beyond histone modifications. HDAC inhibitor panobinostat and another HDAC6-specific inhibitor, tubacin, were shown to cause hyperacetylation of α-tubulin, disruption of the interaction between HDAC6 and dynein, leading to the increase in ubiquitinated proteins (Catley et al., 2006). In another study, LBH589 induced caspase activation, poly-(ADP-ribose) polymerase (PARP) cleavage, and also α-tubulin hyperacetylation. The administration of LBH589 and bortezomib together led to the creation of anomalous bunches of hyperacetylated α-tubulin, albeit with reduced aggresome diameter and apoptotic nuclei. These results suggest that protein acetylation homeostasis can be a factor affecting sensitivity to PIs.

The acetylation is not the only posttranslational protein modification playing a role in PI sensitivity. Inhibition of specific histone-demethylating enzymes can also have a synergistic effect with PI. Lysine (K)-specific demethylase 1 (LSD1) inhibition synergizes with carfilzomib treatment. LSD1 is a histone methylation “eraser” involved in removing methyl groups fromH3K4me1/2 and H3K9me1/2, as well as non-histone proteins, thereby exerting both transcriptional repression and activation and regulating protein stability (Chen et al., 2011; Ziogas et al., 2017). LSD1 silencing enhanced carfilzomib sensitivity in both PI-resistant and -sensitive MM cells, resulting in increased tumor cell death. Moreover, LSD1 inhibitors (SP2509, SP2577, and CC-90011) triggered synergistic cytotoxicity in combination with different PIs in MM and other B-cell neoplasms. Carfilzomib/SP2509 treatment exhibited a favorable cytotoxicity profile toward PBMCs and BMSCs leaving them unaffected. The clinical potential of LSD1-proteasome inhibition was confirmed in primary CD138+ cells of MM patients, and MM xenograft models. Possible mechanisms include alternation of DNA damage response (DDR) and proliferation machinery (Bandini et al., 2023).

The inhibitor of another histone methyltransferase also has shown promising effect in combination with PI. UNC0642 the euchromatic histone-lysine N-methyltransferase 2 (EHMT2) inhibitor displayed a cooperative effect with carfilzomib in MM cell lines, including drug-resistant ones. Carfilzomib/UNC0642 combination exhibited a favorable cytotoxicity profile toward PBMCs and bone marrow-derived stromal cells. To exclude off-target effects it was proved that UNC0642 treatment reduces EHMT2-related molecular markers and that an alternative EHMT2 inhibitor recapitulated the synergistic activity with carfilzomib. EHMT2 inhibition could provide a valuable strategy to enhance PI sensitivity and overcome drug resistance in MM patients (Mereu et al., 2023).

Tazemetostat, an epigenetic inhibitor of EZH2, the catalytic subunit of the polycomb repressive complex 2 (PRC2) that functions as a histone methyltransferase, reduces H3K27me3 levels and enhances the anti-MM effects of bortezomib both *in vitro* and *in vivo* (J. Wang et al., 2022). Interestingly, HRP2 protein, identified through Crispr Cas9 library screening acts as a suppressor of chemoresistance to PIs. HRP2 recognized H3K36me2 and recruited the histone demethylase MYC-induced nuclear antigen (MINA) to remove H3K27me3.

The evidence suggesting the interplay between metabolism, methylation homeostasis, and PI efficacy comes from the studies where IDH2 (isocitrate dehydrogenase 2) was identified as a therapeutic target through Crispr Cas9 screening. IDH2 is an enzyme in TCA cycles that catalyzes the oxidative decarboxylation of isocitrate to 2-oxoglutarate. Unlike in other tumors such as glioma, IDH2 is not frequently mutated in MM (Langer et al., 2010)**.** However, IDH2 emerged as a top target, showing synthetic lethal activity with the PI carfilzomib. The combination of FDA-approved PIs with a pharmacological IDH2 inhibitor (AGI-6780) triggered synergistic cytotoxicity in MM, MCL, and Burkitt’s lymphoma (BL) cell lines. Treatment with carfilzomib/AGI-6780 led to increased death of primary CD138+ cells from MM patients and exhibited a favorable cytotoxicity profile towards peripheral blood mononuclear cells and bone marrow-derived stromal cells. The combination of carfilzomib/AGI-6780 significantly reduced tricarboxylic acid (TCA) cycle activity and ATP levels, via enhanced IDH2 enzymatic inhibition. This occurred through the reduction of nicotinamide phosphoribosyltransferase (NAMPT) expression, thus limiting IDH2 activation through the NAD + -dependent deacetylase SIRT3. The combination of carfilzomib with either NAMPT or SIRT3 inhibitors consistently impaired IDH2 activity and increased the death of MM cells. NAMPT/SIRT3/IDH2 pathway inhibition enhances the therapeutic efficacy of PI (Bergaggio et al., 2019).

### 7.8 Environmental factors

Myeloma cells are exceptionally sensitive to proteasome inhibition and the reason for this state is still under investigation. It is widely accepted that myeloma proteasomes must degrade the larger amounts of misfolded immunoglobins or other proteins these cells produce as compared to normal cells. Therefore, inhibition of proteasome causes protein overload and cell death. Increased body temperature - hyperthermia causes protein degradation and substrate overload on 26S proteasome. Not surprisingly MM cells are prone to heat shock. Unlike other cells, MM sensitive to PI cannot withstand heat shock. Shifting the temperature to only 39°C is enough to augment MM sensitivity to proteasome inhibitors. Thus, mild hyperthermia (e.g., 39°C) may enhance the efficacy of proteasome inhibitors in the treatment of myeloma (Sha and Goldberg, 2020).

### 7.9 Maturation status of MM

Myeloma cells are terminally differentiated plasma cells. Their maturation status is associated with a specific transcriptional signature. Likewise, XBP1s is essential for differentiation of B lymphocytes to plasma cells, and its knockdown induced de-differentiation of cells towards plasmablasts, accompanied by reduced Ig production, expression of UPR genes and reduced ER stress. In consequence, cells with lower level of XBP1s and IRE1 are less sensitive to bortezomib. The appearance of so called “sweet spot” maturation population, in the stage between B cells and PC might be the reason of lower sensitivity to the ER stress induced by PIs. Likewise, bortezomib-sensitive cells have deficiencies in responding by UPR after disturbance of Ca2^+^ homeostasis, due to increased expression of IRE1, sXBP1, GRP78, and pPERK (Leung-Hagesteijn et al., 2013). The upregulation of all three branches of UPR was detected in MM cells resistant to bortezomib and carfilzomib (Kubicki et al., 2022).

### 7.10 Microenvironment

There is a strong interaction between Bone Marrow Microenvironment (BMME) and MM cells. The myeloma BMME is characterized by the presence of many cell types such as osteoclasts and osteoblasts, immune cells, mesenchymal stromal cells and many others. All of these components contribute to the promotion of plasma cell proliferation and the transmission of signals to tumor cells resulting in resistance to treatment. One of the factors contributing to drug resistance and disease progression is release of exosomes by distinct components of the BMME (Pandian et al., 2023). The different cargo of exosomes in MM can clearly differentiate responders from non-responders to PIs. Likewise, miR-16-5p, miR-15a-5p, miR-20a-5p, and miR-17-5p were expressed at higher levels in plasma of MM patients who responded to bortezomib in comparison to those resistant to this PI (L. Zhang et al., 2016). The deep sequencing revealed great amount of both lncRNA and mRNAs differentially expressed in exosomes derived from serum of bortezomib-sensitive MM patients in comparison to bortezomib-resistant. The most enriched pathways within those differentially expressed genes were mTOR, platinum DR, and the cAMP and PI3K–Akt signaling pathways (Tang et al., 2021). CircRNA is a class of non-coding RNA with the circular structure that is also one of the components of exosomes (Han et al., 2022). The circMYC is a circular RNA derived from MYC gene that was found at higher level in MM patients serum-derived exosomes in comparison to the healthy donors, and among MM the lower level of circMYC was associated with better response to bortezomib (Luo and Gui, 2020).

Among BMME-derived factors that could affect sensitivity of PIs in MM are bone marrow mesenchymal stem cells (BM-MSC). BM-MSC produce great amount of soluble factors like TNFα, IL-1β, IL-6, IL-8, and IGF-1. The concentration of those cytokines is increased in MM in comparison to healthy donors (Arnulf et al., 2007; Markovina et al., 2010; Iannozzi et al., 2022). Likewise, the bortezomib resistant MM had higher amount of IL-8 in comparison to the sensitive MM (Markovina et al., 2010).

### 7.11 Metabolic adaptation

Metabolic adaptation is an another important factor shaping the response of cells to proteasome inhibition. This includes alteration in glucose metabolism, pentose phosphate pathway (PPP), mitochondrial pyruvate transport, and serine metabolism.

Hypoxia as an imbalance between increased oxygen demand and insufficient supply is a state of reduced oxygen level. In myeloma, it induces cell dedifferentiation, reduces cell proliferation and induces G1-cell cycle arrest leading to the acquisition of quiescent state. It also promotes tumor progression. The main transcription factor induced by this condition is HIF1 and among its targets the most enriched functional group of genes is associated with glucose metabolism. Under hypoxic conditions the decreased sensitivity of MM cells to bortezomib has been linked to higher glycolytic activity. Lactate dehydrogenase A (LDHA) catalyzes the final step in the glycolytic pathway. Likewise, the depletion of HIF1A and LDHA restored bortezomib sensitivity of MM cells, both under normoxic and hypoxic condition (Maiso et al., 2015).

Among glucose metabolism, also PPP was less active in MM cells sensitive to bortezomib. As multiple proteins involved in PPP pathway are also involved in glutathione (GSH) metabolism, the consequence of decreased PPP activity was the decreased ratio GSH/GSSG (glutathione disulfide) in sensitive cells in comparison to the resistant ones, pointing out the decreased anti-oxidant capacity of PIs sensitive cells (Zaal et al., 2017b). Those cells lack the capacity to regenerate GSH and NADPH, which makes them more sensitive to oxidative stress compared to the resistant cells.

Recently, mitochondrial pyruvate transport was identified as a modulator of proteasome inhibitors in MM cells. Particularly high level of mitochondrial pyruvate carrier complex 1, MPC1 is associated with weaker response to both bortezomib and carfilzomib (Zaal et al., 2017b).

Serine metabolism plays a key role in sensitivity to PIs. Altered serine metabolism was shown to be present in all bortezomib-resistant cell lines tested in comparison to the sensitive ones indicating that it plays an important role in sensitivity to proteasome inhibitor in addition to changes in proteasome. The occurrence of resistance is associated not only with high activity of serine synthesis pathway but also with phosphoglycerate dehydrogenase (PHGDH) overexpression (Zaal et al., 2017b). Lower level of PHGDH was associated with better survival of MM, and its expression increased during the course of the disease. PHGDH promotes proliferation and bortezomib resistance by upregulation of GSH production. Thus, targeting PHGDH may have the potential to overcome resistance to this proteasome inhibitor in multiple myeloma (Wu et al., 2020).

Among the metabolic pathways underlying the acquisition of bortezomib resistance is protein glycosylation, the process that controls essential biological pathways. It appears that bortezomib-resistant MM U266 cells show increased concentrations of glycosylated UDP-derivatives, comparing to bortezomib-sensitive U266 cells. The availability of these compounds ensures post-translational glycosylation of proteins, thus controlling cell signaling, mitochondrial functions, and apoptosis. Higher activity of hexosamine biosynthetic pathway (HBP) that generates UDP-GlcNAc corelates with increased resistance to chemotherapeutics (Liu et al., 2018). Bortezomib-resistant U266 cells show increased expression of O-GlcNAc transferase (OGT) that catalyzes the addition of the O-GlcNAc to proteins and higher concentrations of UDP-derivatives, stabilized, in contrast to bortezomib-sensitive cells, upon bortezomib treatment. Increased mitochondrial-dependent energy metabolism supports the high energy requirements of the HBP pathway and protein glycosylation in bortezomib-resistant cells (Tibullo et al., 2020)

## 8 Conclusion

Cellular response to treatment with proteasome inhibitors is an extremely complex process depending on many factors. The change in apoptotic sensitivity to this group of drugs induced by differences in expression or proteasome burden indicates a cause-and-effect relationship between stress and the apoptotic response. An equally important factor in the response to proteasome inhibitors is serine synthesis and metabolism. This is why the balance between proteasome burden and degradation capacity and also the activity of metabolic pathways are the key determinants of apoptotic sensitivity of cells to proteasome inhibitors. Likewise, the impact of NFkB pathway activity on cells sensitivity to PIs is highly complex and requires further studies. Those should involve the implication of tumor microenvironment factors, like cytokines or presence of other BM cells, as potent modulators of NFkB pathway in MM. One of the intriguing issues, lacking response in the literature up to now is head-to-head comparison of cells response to bortezomib and carfilzomib, as the main proteasome inhibitors used in MM treatment.

Future research should be directed towards a better understanding of the molecular basis of MM cells sensitivity to proteasome inhibitors, which may find application in clinical practice in the future.
